# Synthetic routes to theranostic applications of carbon-based quantum dots

**DOI:** 10.5599/admet.1747

**Published:** 2023-05-08

**Authors:** Pemula Gowtham, Karthick Harini, Anbazhagan Thirumalai, Pragya Pallavi, Koyeli Girigoswami, Agnishwar Girigoswami

**Affiliations:** Medical Bionanotechnology, Faculty of Allied Health Sciences, Chettinad Hospital & Research Institute (CHRI), Chettinad Academy of Research and Education (CARE), Kelambakkam, Chennai, TN-603 103, India

**Keywords:** Bottom-up approach, carbon dots, hydrothermal synthesis, synthetic route, theranostic applications

## Abstract

**Background and Purpose:**

Modern technologies are making advanced paths to address emerging issues. The development of carbon dots (CDs) technology at a tiny level has been researched to have made impeccable strides in advancing the modern scientific field, especially in nanomedicine.

**Experimental Approach:**

Researchers have gained much attention on CDs of their unique properties in the synthesis, easy surface modifications, excellent optical properties, low toxicity, and water solubility. Doping carbon dots with other elements makes them more convenient for their use in the medical sector.

**Key Results:**

The manuscript provides a detailed discussion of the two main methods, including the hydrothermal pathway. CDs are synthesized bottom-up by building up molecules at the atomic scale and top-down by transforming large carbon particles into nanoscale dimensions.

**Conclusion:**

The present article discussed the role, importance, and recent advancements in the synthesis of CDs, by using various approaches giving importance to the hydrothermal process. Recent investigations, their mechanism, and theranostic applications have also been reported.

## Introduction

Carbon dots (CD) are specific quantum dots containing carbon nanocrystals with diameters smaller than 10 nm. Due to their quantum confinement, these ultra-small carbon crystals show photoinduced electron transfer-driven tunable photoluminescence, chemiluminescence, and electrochemical luminescence properties [1]. Compared to organic fluorophores, carbon dots exhibit high tunable flexibility, improved aqueous solubility, biocompatibility, bioavailability, chemical inertness, and considerably low photobleaching to apply in biomedical imaging and biosensing [2,3]. Their high flexibility and biocompatible properties allow these nanosized carbon dots to be highly useful in bioimaging, drug delivery, and diagnosis [4,5]. Owning to the electron transfer mechanism and good optical absorption, CDs are regarded as a good photosensitizer due to their ability to split water molecules and reduce carbon dioxide and pollutants degradation upon irradiation with UV-Vis-NIR region. CDs display photocatalytic properties in the presence of other photosensitizers and redox mediators like enzymes or nanocomposite that functions as electron shuttles in between CDs and enzyme-bound substrates [6,7]. It was reported that CDs, in combination with SiO_2_, Au, Ag, and Cu nanocomposites, are widely used in the oxidation and reduction of alkanes, cyclooctenes, nitrobenzenes, quinones, etc. In semi-biological photosynthesis, the enzyme fumarate reductase catalyzes photo-enzymatic degradation after combining with CDs to convert fumarate to succinate [8].

The CD can be co-doped with other elements, such as N, S, Cl, and Br, and other metal ions, such as copper, titanium, zinc, etc., which improves photocatalytic activities [9,10]. These elemental amalgamations enhance the electron-exchange ability of carbon dots that take a major role in redox reactions at the surface. These induced redox properties are used in wastewater management and hydrogen generation. The graphite nanoparticles lacked molecular dynamics simulation studies. Volker *et al.* designed fluorescent organic molecules whose surface is functionalized with surface-active groups to provide high solubility, including -CO-NH_2_, -COO, -OH, and -NH_2_ [11]. The density-functional theory and configuration-interaction calculations show the structure-activity relationships of QDs [10, 12]. A study reported by Song *et al.* [13] stated that both the ethylene diamine and citric acid precursors undergo a thermal reaction resulting in the formation of bright blue fluorophore products, allocated to understanding the mechanism of photoluminescence in the specific molecular species. Different combinations of different materials with citric acid lead to the formation of different kinds of molecular fluorophore species [13]. The study by Zhou *et al.* involves the preparation of the most refined form of high fluorescent graphite carbon nitride QDs with urea and sodium citrate under high temperature in an autoclave, resulting in the formation of surface decorated with negatively charged oxygen-rich surface active groups [14].

## Synthesis mechanism of carbon dots

**Figure 1.**
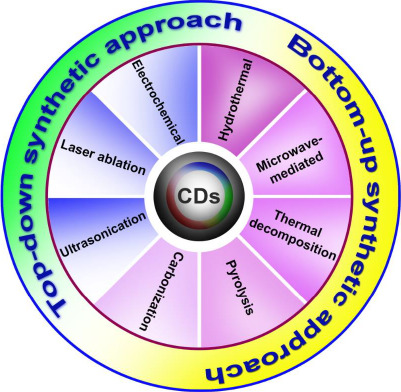
Different approaches for carbon dot synthesis

There are two main approaches to synthesizing carbon dots: top-down and bottom-up (Figure 1). Laser ablation techniques and electrochemical methods are classified as more popular top-down approaches. In this method, the micron-sized carbon structures divide into (or) break down into nanosized crystals ranging less than 10 nm. The preparation of blue luminescent nanocrystals via the electrochemical method was reported by Zhou *et al.* [15], for which multi-walled carbon nanotubes (MWCNTs) were used as the precursor. The laser ablation technique is generally used to amalgamate nanoparticles in the liquid medium by modifying solid particles using laser pulses. This made it convenient and understandable for various laser parameters such as irradiation time, pulse duration, and wavelength in the solid and solvent features. Reyes-Contreras *et al.* [16] synthesized carbon nanoparticles with the size ranging from 4 to 20 nm using acetone as a liquid media by targeting graphite with a pulse of neodymium-doped yttrium aluminum garnet (Nd-YAG) laser pulse with an emission wavelength of 1064 nm. The excitation wavelength can be altered to covert this from blue to yellow. The nanosized crystal structures are synthesized via polymerization reaction or conversion of suitable atoms and molecules into large nanostructures using different synthesizing procedures such as microwave-mediated synthesis techniques, hydrothermal, pyrolysis synthesis, ultrasonication method, *etc.*, classified under the bottom-up approach. Zeng *et al.* [17] fabricated green-emitting carbon dots with a size of 2-6 nm utilizing citric acid and urea by microwave synthesis. CDs are showing exuberant in fields of imaging and delivery, yet still, it is very important to synthesize the particle to attain a uniform shape and size.

### Top-down synthetic approach

#### Electrochemical procedure

The electrochemical method is the heterogeneous method of synthesizing nanoparticles with a highly efficient and controllable process to obtain the purified and controllable particle size and shape. The preparation of CDs using this method utilizes the chemical cutting process of the carbon material. This technique mainly obtains a high quantum yield, higher purity, and cost-affordable CDs. Deng *et al.* [18] synthesized the red and blue CDs by the electrochemical method utilizing ethanol, which is low molecular weight alcohol. OH plays a major role in obtaining the CDs with high quantum yield in alkaline conditions NaOH/EtOH. The synthesized particle sizes were 2.1, 3.5, and 4.3 nm, and the particles were confirmed with a peak at 23° in XRD [18]. Qingxiao *et al.* [19]. synthesized CDs by an electrochemical method using o-phenylenediamine (OPOD) as the carbon source During this process, it undergoes many changes to obtain the yellow color emission of CDs at the platinum anode [19]. Ray *et al.* [20] synthesized the CDs using nitric acid oxidation of carbon soot, having a nanocrystal size ranging from 2-6 nm, by applying a temperature of 100 °C for 12 hours. Qiao *et al.* [21] synthesized fluorescent carbon nanocrystals with a uniform size of 1.9±0.3 with blue color and 3.2±0.5 with yellow color using the graphite electrode and calomel electrode in NaH_2_PO_4_ as medium. Liu *et al.* [22] synthesized the CDs using the electrochemical method with an average size of 4.0±0.2 to help in the detection of the Fe^3+^ ions in tap water. At room temperature, colorless CDs convert to yellow upon oxidation. Photoluminescence quenching of CDs occurs due to the functional groups' presence on the CDs, which helps the Fe^3+^ to bind with the outer layer of carbon dots, leading to quenching of the compounds [22]. Niu *et al.* [23] prepared nitrogen-doped CDs using an electrochemical method applying pyrocatechol as the precursor for the detection of alkaline phosphatase and pyrophosphate anions. This method does not require harsh reaction conditions like higher temperatures or toxic chemicals; at the same time, good crystallinity of CDs can be maintained. In this method, the yield of particles is very low due to intercalation, and the process is very slow due to the accumulation of ions.

#### Laser ablation

The laser ablation technique is used for the breakdown of bulk materials into simple nanoscale materials, which are present in the liquid or gaseous environment using the laser beam on the targeted material, which results in the formation of fine ablation of materials [24-26]. Laser ablation is the technique that uses light sources to excite the atoms from the lower excitation region to the higher excitation region, and the atoms trigger the initial process for the formation of crystals. Material ablation occurs when the material and the laser interact due to the coupling of electron and photon interactions with the free valency of electrons reaching the higher excited state. The size of the obtained materials can be tuned by controlling the parameters such as pulse repetition rate, pulse width, energy, and solubility factors for the formation of particles of different sizes [27]. Carbon dots obtained by immersion of graphite in a liquid medium of ethylenediamine or poly(ethyleneimine) using the Nd:YAG laser obtained a particle size of 1 to 3nm, which is spherical in shape, was synthesized by Kaczmarek *et al.* [28].

Reyes *et al.* [29] synthesized the carbon dots targeting the carbon source in the acetone medium by inducing the nanosecond laser pulses of Nd:YAG laser, which was targeted to the specific part with the different wavelengths by using the infrared lens and UV-Visible lens. Nguyen *et al.* [30] synthesized blue and red CDs using the ablation technique and an infrared laser with 800 nm in polyethylene glycol as the medium to obtain CDs from graphite powder. The size of the CDs depends on the laser dependence, and it emits high photoluminescence (PL) when treated with low laser influence upon them. The size of the carbon dots is inversely proportional to the irradiation time [30]. Yu *et al.* [31] synthesized CDs with toluene as a precursor in the presence of the Nd:YAG laser with the wavelength produced in different sizes by slightly different ranges in the pulse. Formation from the higher was used for obtaining the nano-size particles from the large compounds by using a laser source that stimulates and triggers the photons to be used as energy for the amplification. The preparation of CDs by this method has potential photoluminescence properties that are highly stable, tunable, and bright are the advantages. The disadvantages include complex procedures, low output, and the size distribution of the particles that are not uniform. The process needs additional passivation, oxidation, and irradiation to control the products.

#### Ultrasonic treatment

The ultrasonic treatment mechanism is characterized by the frequency of ultrasound waves generated in the liquid medium, known as acoustic cavitation. When these waves pass in the liquid medium leads to generate microbubbles with high intensity to create the local hotspots by generating an inertial effect by continuous longitudinal waves and refractions. Obtaining high pressure and temperature in the surroundings enhances the molecular breakdown of the compounds [32]. The ultrasonication method of synthesizing the CDs was mainly dependent on the parameters like sonication time and the amplitude for obtaining the specific size. Kumar *et al.* [33] synthesized yellow CDs ranging between 2-9 nm using polyethylene glycol by one-step synthesis. The particle size varies as the sonication amplitude varies, and from which CDs of different sizes can be obtained. As the sonication amplitude increases, emission intensity and curve increase [33]. Dehvari *et al.* [34] synthesized nitrogen-doped carbon dots as a probe using a crab shell by ultrasonication irradiation. The one-step sonochemical approach produced a product with a particle size of 8 nm and 14.5 % of fluorescence quantum yield. The product was highly soluble with functional groups over the surface. Due to the targeting moiety, it featured both therapeutic and diagnostic applications [34]. Li *et al.* [35] used a one-step ultrasonic method for synthesizing blue CDs ranging from the natural precursor. The size range was around 5 nm. The particle exhibited theranostic potential due to high loading capacity and enhanced photoluminescence properties [35]. Wang *et al.* [36] synthesized upconversion CDs using BiSbO_4_ via an ultrasonication process to enhance the photocatalytic properties and chemical stability by redox properties, which would, in turn, improve the degradation of organic pollutants. Studies conducted on the top-down mediated synthesis of CDs are compiled in Table 1. Ultrasonic treatment is a low-cost and green method to produce nanomaterials that have the disadvantages of getting ultrasonic reactors for the large-scale production of CDs.

### Bottom-up Approach

#### Microwave-mediated synthesis

Microwaves range from 0.3-300 GHz, where microwave uses the heating theory for the organic reactions, which occurs in closed reactors using dielectric heating effects that accelerate the rate of chemical reactions [44]. The organic molecules mainly depend on the polarity to absorb the heat energy and to convert the molecules or the distribution of electric charge over the atoms. A dielectric material contains either permanent or induced dipoles when suspended between two electrodes acting as capacitors. The materials have either a positive or negative charge that is stored on the surface of the molecules, which undergo molecular rotation when irradiated with the microwave [45]. The microwave-mediated synthesis has advantages compared to conventional heating synthesis as it gives rise to more heating capacity when molecules undergo a chemical reaction. The conventional method needs a long period of time to make the reaction medium reach the required temperature, and at times it may also lead to the degradation of the reactants. In contrast, microwave-assisted synthesis offers a fast reaction time through bypasses the heating of microwaves on the reaction vessel. Microwave-assisted synthesis of carbon dots has surface modification with different solubilized functional groups such as carboxyl, hydroxyl, or amines, making them highly soluble. Some of the heterocyclic aromatic amines are known to be carcinogens. Lopez *et al.* [46] synthesized the CDs using the lactose by microwave process, where HCL is used as precursors and given a microwave treatment for 15 min. The transmission electron microscope (TEM) was used to determine the size of obtained CDs and to study the details of functional groups on the surface. The CDs were around 10 nm in size, while the surface shows -OH and C=O functional groups. These functional groups account for excitation-dependent fluorescence with maximum emission at 450 nm and excitation at 350 nm with an intensity of 13 % [46]. Zhao *et al.* [47] worked on the synthesis of CDs using ethylenediamine as a nitrogen dopant via a microwave-mediated method by heating the solution in a microwave oven for around 10 mins. TEM analysis of the CDs revealed the size range, about 2-5 nm, and then the surface groups that showed the presence of O-H and N-H, C-H, C=O, and C-N functional groups, contributing to water dispersibility. The maximum emission and excitation spectra were 450 and 370 nm, respectively. As the pH values increase, the fluorescence intensity of CDs decreases, and the presence of metal ions such as Co^2+^ shows a brown-colored CD. At the same time, it remains the same for the other metals, which helps in the detection of water samples [47]. Xu *et al.* [48] synthesized CDs doped with sulfur and nitrogen using glycerol with one pot reaction mechanism, a high dielectric constant as precursors, and cystine as the source was heated in a microwave oven for 6 mins. A size range from 1.5-5.5 nm was obtained with the hydroxyl functional groups on the surface, leading to an aggregation-induced enhancement effect resulting in the real-time detection of Hg^2+^ [48]. This method of processing CDs involves no contact heating since the electromagnetic field interacts directly with molecules to create energy. In addition, CDs are produced with a high yield due to lesser reaction time, environmental friendliness, uniformly distributed heating and energy saving modes. It is generally not possible to use microwave reactors for large-scale reactions, since they are confined to small volume. A low boiling point solvent is not suitable in this procedure due to the pressure and temperature limitations.

#### Thermal decomposition

Thermal decomposition is an endothermic reaction that generally uses heat as the reactant for the breakdown of chemical compounds into molecules, and this technique is used to synthesize CDs through the bottom-up method. The major benefits of this method are less time taking process with inexpensive, efficient, and large-scale manufacturing [4].

Ludmerczki *et al.* [49] fabricated luminescent CDs with citric acid and its intermediate derivates, which helps produce highly fluorescent CDs. Here, citric acid monohydrate was heated to 180 °C, and the sample was collected from colorless to light yellow to orange-yellow. The surface modification was done using 3-(aminopropyl) triethoxysilane (APTES), which helps in pH shifting, usually from acidic to basic, and obtained a peak at 450 nm, and the size of the particle was around 5-15 nm [49]. Wan *et al.* [50] utilized a thermal method to synthesize CDs with 1-butyl 3-methyl bromide imidazolium and I-cysteine at 240 °C. With the AFM study, the height of CDs was found to be around 1.0-3.5 nm. This method was used to determine the CDs from the small organic molecules [50]. The method is easy to perform, and the physicochemical properties, including optical properties, can easily be tuned. The formation of impurities during the decomposition process can affect CDs' quality, which needs further purification.

#### Carbonization synthesis

**Figure 2.**
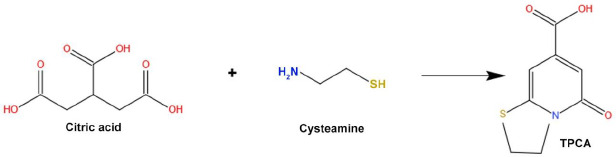
Schematic representation of the mechanism of TPCA formation

The carbonization process is adopted to synthesize large carbon content with solid residues formed from organic materials. This reaction usually takes place in an inert condition through pyrolysis. The carbonization process is the conversion by progressive heating of the organic macromolecular system to a macro-atomic network of carbon atoms. Molecular precursors undergo carbonization, which is the best, cheapest, and one-step process for synthesizing CDs [4]. Shi *et al.* [51] synthesized N, S doped CDs via carbonization technique. This procedure involves the reaction of citric acid and L-cysteine to form TPCA (5-Oxo-3,5-dihydro-2*H*-thiazolo[3,2-*a*]-pyridine-7-carboxylic acid) as a primary component. TPCA is responsible for the production of CDs, which emit strong fluorescence with a high quantum yield (Figure 2).

**Figure 3.**
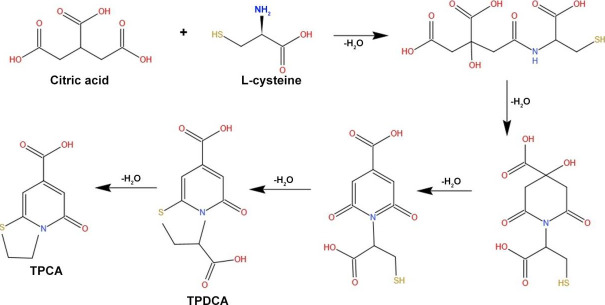
Schematic representation of TPCA formation using citric acid and L-Cysteine as the precursor

Lin *et al.* [52] synthesized high fluorescence quantum yield CDs from L-cysteine and citric acid with a size ranging from 2-4 nm, which helps increase fluorescence for cellular imaging (Figure 3).

Qin *et al.* [53] synthesized CDs using uric acid by carbonization method. The obtained CDs ranged from 1.3 to 7.9 nm with an excitation wavelength of 350 nm, followed by an emission wavelength of 402 nm with a quantum yield of 52.06 % [53]. Using citric acid as a medium, Wang *et al.* [54] synthesized the blue luminescent emitting carbon dots with a range of 4.8-9 nm by thermal reduction. This enhances the quantum yield compared to the normal non-reacted CDs, which were analyzed by a thermogravimetric analyzer [54]. Carbon dots are uniformly formed using a controlled carbonization reaction, and unlike other synthesis methods, they do not make many impurities. This method needs a longer reaction time, and CDs yield is also very low.

#### Pyrolysis synthesis method

The pyrolysis method is also a type of thermal decomposition where the chemical transformation of a sample occurs on heating to high temperatures in an inert environment condition. It is an irreversible process where the organic materials undergo decomposition under high temperatures and controlled pressure. During this process, the organic materials undergo both physical and chemical changes resulting in the formation of carbon residue. Ma *et al.* [55] developed blue-emitting CDs doped with nitrogen using citric acid and ethylenediamine at 170 °C through the pyrolysis method with an average diameter of 2-6 nm in the range with a quantum yield of 88 %. Rimal *et al.* [56] fabricated CDs via the pyrolysis technique of bottom-up synthesis using oleic acid as an organic substrate at 260 °C. The obtained CDs were in the range of 5-10 nm with a quantum yield of 50 %, which also combines with an acetate polymer matrix to form polymer composites that are stable at high temperatures [56]. Using glycerol solvent as the precursor, a new type of CD was synthesized using the pyrolysis method, where it was heated up to 230 °C, ranging from 2-4 nm. The prepared CDs were passivated by polyethylene glycol (PEG), which increases biocompatibility. The thermogravimetric analyzer was used to identify the heat loss, and the intensity was recorded as 14.3 %, as studied by Lai *et al.* [57]. Tang *et al.* [58] synthesized blue-emitting CDs using lactic acid ionic liquid via a one-pot pyrolyzing technique. According to TEM results, obtained CDs ranged from 4.4 nm and the emission of 454 nm and excitation of 365 nm, which gives an intensity of 16 % [58]. Studies that have been conducted to synthesize CDs via bottom-up approaches are compiled in Table 2. The pyrolysis method is cost-effective and does not need high-quality reagents and arrangments. The morphology and the size of CDs can be controlled easily by varying the reaction conditions. Scaling up the synthesis of CDs and determining the growth temperature are the major issues of this method.

#### Synthesis using hydrothermal method

**Figure 4.**
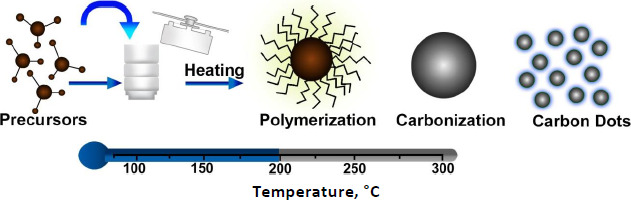
Schematic representation of the mechanism of Hydrothermal mediated carbon dot synthesis

Hydrothermal synthesis is referred to as synthesizing of nanoparticles using chemical reactions in an aqueous medium at high temperatures. Generally, hydrothermal synthesis of chemical reactions occurs in a closed autoclave with high temperatures (100-1000 °C) and pressure (1-100 MPa). The hydrothermal synthesis interfaces with solids and solutions to form nanoparticles during phase transfer. The separation mechanism mainly depends on the reactants, chemical reactions, conditions, and the relation between the structures and properties of the compounds [69]. In hydrothermal synthesis, the reaction mechanism mainly depends on the reactant molecules that react with the solution giving rise to different products with different structures even if the same reactants were used [70,71]. This is also very useful in the chemical combination of compounds that cannot be done using solid-state synthesis. The particles synthesized using this method have unique physical and chemical properties due to their aggregation, condensed state, valance state, and structure, making the compound grow in a single crystal with controlled size and structures (Figure 4). Depending on the temperature used in the hydrothermal synthesis, they are again classified into subcritical and supercritical synthesis. The subcritical synthesis reaction occurs between 100 and 300 °C, whereas the supercritical synthesis reaction goes up to 1000 °C [72,73]. Different organic solvents have different soluble properties, which change due to high temperature and pressure. The hydrothermal system mainly accelerates the reaction rate and intense the hydrolyzation reaction, which leads to changes in the redox potential of the reactants. The medium used in hydrothermal synthesis plays a major role in the obtained products, as various organic solvents dissolve the reactants and form a solvent-reactant complex [74]. This affects the rate of the chemical reaction, and hence, selecting the organic solvent is critical since it plays an important role in the reaction process. The solvent can be selected based on molecular parameters such as melting point, boiling point, density, molecular volume, rate of evaporation, dielectric constant, molecular weight, and solvent polarity. The hydrothermal method has become the most important technique for the fabrication of nanomaterials, such as nanohybrids and nanocomposite materials.

The nanoparticles synthesized using this method possess chemical homogeneity and solubility with monodispersed size and shape. Researchers developed many strategies for obtaining the desired nanostructures by controlling the hydrothermal conditions (Table 3). Many factors important in obtaining the desired structures are internal factors such as the nature of reactants, concentration, pH value of the reaction system, and pressure of the reaction system. Other external factors, such as reactive time and temperature, also reflect in the structural aspect [74]. Hydrothermal synthesis of CDs is cost-efficient and easy to synthesize from different components such as amines, saccharides, organic acids, and their derivatives [4]. CDs are modified with various chemical components to enhance their photoluminescent properties. Photoluminescence mainly depends on two major principles: 1) the carbon core with modified functional groups and 2) the surface state, which is determined by the hybridization of the carbon backbone and connected chemical groups. In the hydrothermal method, various precursors have a dominant role in synthesizing CDs to obtain different sizes and colors when doped with surface passivating agents. Based on the surface passivated compounds, the absorption properties vary from one CD to another. CDs are prepared with many precursors, including citric acid, urea, sodium citrate, ethylenediamine, and glucose. Generally, the organic precursor will be taken in an autoclave and reacted with hydrothermal reactors at high temperatures [8]. The properties of CDs depend on carbonization, an endothermic process that relays directly on the temperature. The temperature should not be too high or low, as the high temperature degenerates the surface structure due to over-oxidizing the molecules, whereas the low temperature leads to the formation of defective CDs since the optical properties directly depend on the reaction time and temperature [5,75]. This part will discuss various studies involving a hydrothermal technique for synthesizing CD with a minimal size range and high quantum yield for various biomedical applications.

**Figure 5.**
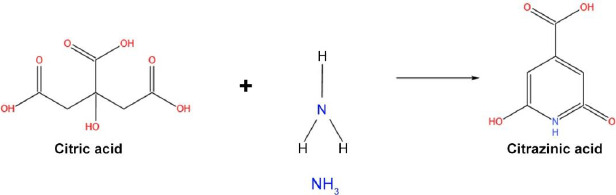
Schematic representation of the reaction of citric acid with ammonia

Fluorescent CDs have recently gained tremendous attention, yet there is no proper evidence to support the origin of fluorescence in CDs. Citric acid is a more commonly used precursor in synthesizing carbon dots [76,77]. Citric acid, when treated with ammonia, forms fluorophore citrazinic acid through a hydrothermal reaction process. The citrazinic acid derivatives play a major role in the photoluminescence property in CDs. A study reported by Schneider and team used citric acid and hexamethylenetetramine (HMTA) for the preparation of CDs via hydrothermal reaction, which released ammonia at high temperatures and formed citrazinic acid derivatives fluorophores [78]. But the obtained CD-HMTA compounds exhibited low fluorescence, which was explained in terms of the decomposition rate and strength of nucleophile (NH3) (Figure 5). Both characteristics decide the fluorescence of a compound, and they are directly proportional.

**Figure 6.**
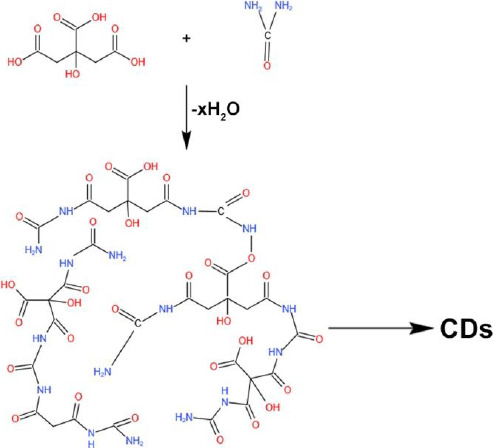
Schematic representation of the reaction of citric acid and ammonia to form CDs

Many reports drew a path suggesting that fluorescence CDs peak can be varied by changing the excitation wavelength range and forming different emission paths, which was unclear. CDs can be passivated with different functional groups, such as carbonyl and hydroxyl groups, which can be used as trapping agents, making CDs emit various colors in different excitation wavelength ranges. Temperature plays a major role in the surface groups' passivation and emitting fluorescence. In other cases, the emission also occurs through a radiative transition of sp^2^ carbons in the amorphous sp^3^ matrix. The CDs with the rich source of amino-group are desirable for their controlled surface passivation and thus exhibit excitation-independent fluorescence due to the surface-coated functional groups [79]. Zholobak and team fabricated CDs with urea and citric acid as a precursor in a ratio of 1:3 at 133 °C for the formation of blue emission and green emission CDs, where the blue emission CDs were obtained from the citrazinic acid and the green emission was due to the formation of citrazinic amide [80]. Urea acts as a carbon source and as a surface amino functional group on the surface of CDs and can contribute to changes in the luminescence properties. Kasprzyk *et al.* [81] obtained CDs using citric acid and urea from the open and closed reaction systems. Ammonia produced during this reaction plays a major role in the emission of CDs (Figure 6 ).

**Figure 7.**
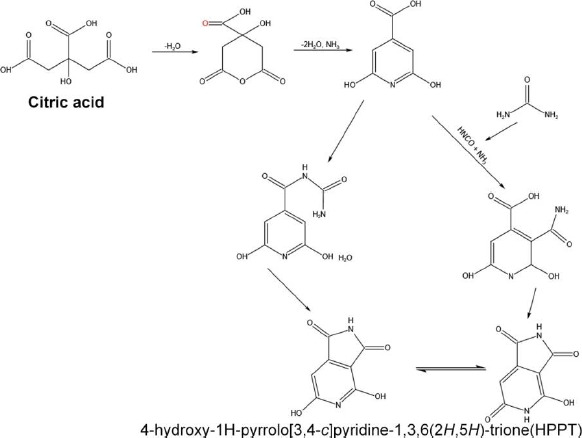
Schematic representation of the mechanism of HPPT formation

The deeper and more detailed information about these CDs' chemical structures and surface states has been studied using NMR spectroscopy and electrospray ionization mass spectrometry. This study confirms the correlation between the shifting of pyridone nitrogen and imide nitrogen with hydrogens in fluorophore structures. The interpretation of the chemical structure of the green fluorescence particle formed 4-hydroxy-1*H*-pyrrolo[3,4-c]pyridine-1,3,6(2*H*,5*H*)-trione (HPPT), which was first reported by this group [82]. HPPT can be formed by using two main pathways (Figure 7). One possible formation involves citrazinic amide or acid from citric acid and urea. Further, the urea was broken-down to obtain isocyanic acid, which was then added to the pyridine ring at position 3. The HPPT was then formed after closing the imide ring. The other route follows the creation of an intermolecular complex of amide between citrazinic and acid urea. After the formation of intermolecular amide, intramolecular condensation and cyclization occurs by excluding ammonia [82]. In the first mechanism, isocyanic acid is obtained from urea by the thermal-induced mechanism in the presence of water. In the second mechanism, an end product citrazinic acid, with a blue emission, is formed due to the reaction between citric acid and urea in an aqueous medium. It was then transformed into green-emitting HPPT after the evaporation of water. In both mechanisms, citrazinic acid is a byproduct to form HPPT, whereas it can also be obtained directly by heating urea and citrazinic acid [82].

**Figure 8.**
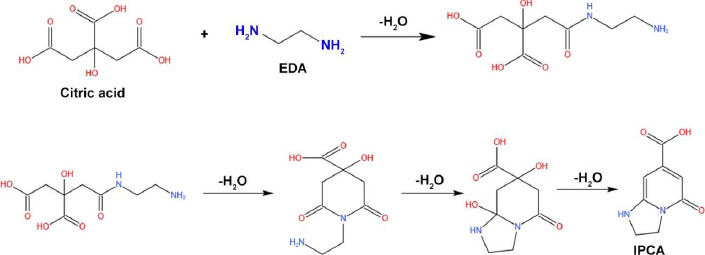
Schematic representation of the reaction of citric acid and EDA to form IPCA

Song *et al.* [13] worked on the synthesis of fluorescent CDs prepared with precursor such as citric acid and EDA. The team utilized the hydrothermal method by heating the solution at a constant temperature of 140 °C for 10 h. The prepared CDs ranging from 2-6 nm led to the formation of fluorescence molecules (1,2,3,5-tetrahydro-50oxo-imidazo[1,2-*a*] pyridine-7-carboxylic acid, IPCA) via amidation reaction to form the branched polymers. Further, it undergoes a carbonization reaction to obtain the amorphous matrix (Figure 8) [13].

Liu *et al.* [83] fabricated CDs using metronidazole through a hydrothermal approach by heating them to 250 °C for 8 h. The prepared CDs attained a size of 2.9 nm with a quantum yield of 28.1 %. The nitrogen-rich functional groups on the surface were responsible for antibacterial activity resulting from the inhibition of the growth of obligate anaerobes and favored variegated bioimaging [83].

Carbon dots were formed using citric acid and ethanolamine, which forms derivatives of citrazinic acid and exhibits high fluorescence due to the organic functional groups passivated on the surface of CDs [84]. The photoluminescence behavior of the amorphous mixture containing sp^2^ and sp^3^ carbons supports the photogeneration of electron-hole pairs that induces the radiative recombination of localized trap carriers which locks the sp^2^ clusters bounded by sp^3^ defects and makes the mechanism remains stable in the presence of heteroatom groups. Wang *et al.* [84] synthesized blue-emitting nitrogen-doped CDs using ethanolamine ionic liquid as the precursor via the hydrothermal method by applying the temperature of 200-240 °C for 4 h. The dispersion was dialyzed using 1000 Da for three days to obtain a particle size of 3.4 nm with rich hydroxyl and nitrogen-rich functional groups on the surface with a quantum yield of 24.7 %. The fluorescent probes were useful for detecting heavy metals in tap water [84]. Li *et al.* [85] synthesized the CDs using citric acid with polyethyleneimine via a one-pot hydrothermal reaction for the detection of the Moraceae family compound called morin for double mode detection in human urine. The quantum yield of obtained CDs was recorded as 48.3 %, emission range of 459 nm, and a size range from 3.0 to 6.0 nm when heated at 110 °C for 2 h [85]. Lin *et al.* [86]synthesized CDs doped with phosphorus and nitrogen using citric acid and o-phosphorylethanolamine by hydrothermal method for detecting cadmium ions in serum and urine samples based on the chelation-enhanced fluorescence (CHEF) mechanism. The preparation involved heating up to 180 °C for 12 h, and it was further filtered using a membrane filter to obtain a particle size of about 1-2 nm [86]. A study by Wang *et al.* [87] developed CDs with hydrochloric acid as a catalyst using the combination of L-tryptophan and L-phenylalanine at 200 °C for 2 h with a quantum yield of 21 %. The obtained particle measured about 4.8 nm, and the surface was passivated with hydrazine functional groups and emitted blue fluorescence at 435 nm [87].

Liu *et al.* [88] synthesized blue-emitting nitrogen-doped CDs using citric acid and ethylenediamine as precursors, which emit fluorescence at 465 nm. The mixture was heated to 150 °C for 2 h. The hydroxyl groups and carboxyl groups on the surface induced a negative charge. The quantum yield was recorded as 58.6 %, and the size of the particle was found to be 6 nm. The compound was synthesized as a chemosensor to detect metals in water samples [88]. Another group studied the impact of temperature on the fluorescence emission range. The procedure utilized folic acid and sodium hydroxide. The mixture was then heated at a different temperature from 170 to 200 °C for 5 h. As expected, the fluorescence emission shifted from 447 to 462 due to temperature variation. The optimum temperature used was around 190 °C. The carbonization rate decreased due to a decrease in temperature. The particle size ranging from 3-6 nm was confirmed by using transmission electron microscopy [89]. Wang *et al.* [90] synthesized CDs hydrothermally, using catechol as the carbon source. In the presence of water, the compound was heated to 200 °C for 24 h and placed in a dialyzed membrane to obtain the nanoparticles in the 4.7 nm range. The surface of CDs was passivated with the residual catechol, carboxyl, and hydroxy functional groups, and the maximum emission of the particle was recorded at 345 nm with a quantum yield of 32 %. This work was done for the identification of hypochlorite in the water samples [90]. The carbon dots can also be synthesized via the green synthesis technique. Atchudan *et al.* [91] worked with this idea and synthesized CDs using banana peel with water as the solvent. The procedure involves an autoclave and heating up to 200ºC for 24h. The obtained particles emitted blue fluorescence at 365 nm and size was measured at about 5 nm with a quantum yield of 20 % [91]. The same group reported the green synthesis of CD using the juice of *Phyllanthus emblica* (Indian gooseberry). The nitrogen-doped blue emission CDs were obtained when heated for 12 h at 200 °C. The emission maximum was seen at 411 nm with a particle size of 5 nm, and a quantum yield was calculated as 13.5 %. The obtained compounds were used as fluorescent ink in bioimaging and drug delivery applications [92].

## Applications of CDs

**Figure 9.**
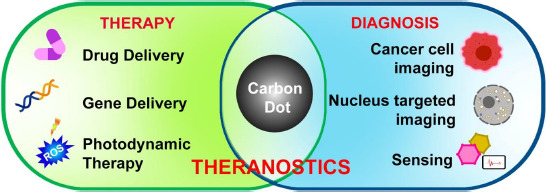
Major biomedical applications of carbon dots

Nanoparticles are gaining interest for their multimodal applicational approach in healthcare industries [114-116]. They are mostly used to formulate therapeutics and imaging agents to improve bioavailability and reduce toxicity [117-120]. Carbon dots, with their high potential characteristic features such as biocompatibility, photostability, and easy surface modifications with high penetration into the cell surfaces, make them great alternatives to the current marketing drug carriers [5]. For the targeted imaging of cells and their structures, CDs are prepared by surface modifications based on the specific targeted molecules to avoid immune responses to prevent them from detection as foreign bodies by the immune system (Figure 9). These surface passivation of CDs helps *in vitro* and *in vivo* applications.

### Cancer cell imaging

If left undiagnosed, cancer will lead to metastasis and death in a short period. As a consequence, it is very necessary to develop novel methods for identifying the early diagnosis of cancer. Carbon dots were considered future candidates for imaging applications due to their unique solubility and low cytotoxicity properties. The surface-modified CDs have the potential to pierce into various types of cancer cells by giving fluorescence imaging. Several studies have reported the preparation of CD for diagnostic applications. Zhou *et al.* [14] synthesized carbon nitride quantum dots using urea and citric acid, which were incubated with the HEK-293T cells and monitored under the confocal microscope. Red and green fluorescence was absorbed in the membrane and cytoplasmic areas and was excited at 543 and 488 nm [14]. Zeng *et al.* [17] synthesized carboxyl-rich surface green-emitting CDs, which showed high fluorescence in HepG2 tumor-bearing mice. This fluorescence also helped to deliver the drug and perform localized therapy [17]. Zhong *et al.* [121] synthesized orange luminescent carbon dots using 1,2-benzenediamine and carbamaldehyde. The mixture was heated at 180 °C for 3 h, and the surface was passivated with the carboxyl groups, which react with the human lung cancer cells (A549). Another study by Lai *et al.* [57] used glycerol via a pyrolysis process to synthesize the CDs surface modified using poly (ethylene glycol). This study used HeLa cancer cell lines incubated on a cover slip and observed under a confocal microscope at the wavelength of 405 nm. The cytoplasm exhibits blue fluorescence, which shows new paths for cellular imaging [57]. Wang *et al.* [122] developed CDs from beer for visualizing the MCF-7 breast cancer cell lines under a confocal microscope with excitation of 405 nm laser. There was no fluorescence in the control group, and a blue color appeared in the cell cytoplasm. The control group showed no fluorescence, and a blue color appeared in the cell cytoplasm. The nucleus was visualized in the range of 543 nm [122]. Liu *et al.* [123] synthesized CDs using folic acid as precursors to obtain a high quantum yield and target cancer cells. This helped in the receptor-mediated cellular uptake in HeLa cells as it binds to the surface and gives a high fluorescence intensity compared to the other cell lines. This study supports that CDs can effectively target cancer cells by folate receptor-mediated processes [123].

### Nucleus-targeted fluorescence imaging

The nucleus is the major component of the cell as it carries hereditary information. Visualization of a nucleus is more important to determine the structure and metabolic features of the cell. Certain functional groups can be decorated on the surface to target and bind to specific molecules, tenable active targeting. The first study reported by Datta *et al.* comprised synthesizing of CDs with a positive charge and allowed them to combine with graphene to form a hybrid nanomaterial [124]. This helped in easy penetration into NIH/3T3 cells through electrostatic interactions. In addition, Ci *et al.* [125] synthesized nitrogen-doped carbon dots using ascorbic acid and poly(ethylenimine) via one-pot hydrothermal synthesis. The temperature here played a vital role in obtaining appropriate minimal particle size. The CDs were co-incubated with HeLa cells in different concentrations and observed under the confocal microscope using CCK8 assay. Due to the presence of zwitter ions on the surface, the CDs can easily penetrate the nucleus by leaving minor parts in the cytoplasm and other organelles. This shows the potential of the CDs to be used as nucleus labeling [125]. Another study reported by Jung *et al.* [126]used beta-alanine as the zwitterionic agent on the surface of CDs prepared with citric acid. The particles were able to target the nucleus, as the zwitterionic surface was more capable of being resistant to the non-specific adsorption of proteins and increased the cytoplasmic uptake and delivery to the nucleus region [126]. Hill *et al.* [127] synthesized green emissive fluorescence CDs using glucosamine and m-phenylenediamine via microwave synthesis procedure. 2,5-deoxyfructosazine was used as a passivating agent to target the nucleus of HeLa cells and human dermal fibroblasts. After the cells are internalized with CDs, LED-mediated selective killing takes place. The interaction with the DNA causes an increase in intracellular temperature, leading to the breakdown of ATP [127]. A green synthesis method reported that blue fluorescence emitting CDs were prepared using citric acid and barley leaves. PK-15 and HeLa cell lines were used for cellular imaging. The results show that negatively charged CDs are observed in the cytoplasm when the excitation is 405 nm, and the neutral CDs are spread over the entire cell, including the nucleus. This study suggests that charge has a specific role in targeting organelles [128]. Mitochondria, the powerhouse of the cell, help produce ATP. The abnormalities in mitochondria may lead to many fatal diseases. CDs have great potential to track cellular fate and mitochondrial disorders. A novel development of functionalized CDs shows high permeability, solubility, and photostability. Sun *et al.* [129] synthesized fluorescent carbon dots by microwave-assisted synthesizing with citric acid and N, N-dimethylaniline. The CDs were labeled on HeLa cells and visualized under the confocal microscope with a laser wavelength of 488 nm. Electrostatic interactions play a vital part in assembling the cationic charge of CDs in the mitochondrial membrane, which has a high negative charge potential. When the mitochondria membrane potential (MMP) decreases, the CDs migrate into the nucleus due to the interactions with nucleic acids [129].

### CD as sensing materials

Carbon dots possess chemical stability, tunable surface functional properties, low toxicity, high solubility, and low-cost efficiency. All these properties make CDs a great candidate in the biosensing field. When the CDs having excellent fluorescence properties are loaded with biosensor molecules, their tunable surface properties help to incorporate different functional groups, in turn, helps in the detection of specific biomolecules and shows excellent advantages in biomedical applications. Zhang *et al.* [130] worked on the synthesis of CDs using citric acid for the detection of Fe^3+^. The nitrogen doped-CDs show fluorescence quenching by non-radiative electron transfer, which can effectively detect Fe3^+^. The detection limit was found to be 2.5 nM [130]. Wang and team [131] synthesized CDs hydrothermally with m-aminobenzoic acid for the identification of Fe^3+^ in water samples. The surface functional groups directly correlate with Fe^3+^ ions, and the detection limit was recorded as 0.05 μm [131].

Mohammadi *et al.* [132] synthesized CDs loaded with DNA. Citric acid and ethylenediamine were used as the precursors for the synthesis. The surface was passivated with DNA probes for targeting the microRNA-155 in serum samples. The assay undergoes the FRET mechanism, and the detection limit was 0.1 aM. This study was conducted by using MCF-7 human breast cancer cell lines to measure microRNA expression [132]. Shan *et al.* [133] synthesized CDs for the identification of hydrogen peroxide (H_2_O_2_). The surface of CDs contains peroxidase-like activity for the identification of H_2_O_2_, and the same can be used for glucose detection at the detection limit of 10.0 mM [133].

### Drug/gene delivery

Carbon dots are used as potential drug carrier vehicles to the target site by making a slight modification over the surface of CDs, and the pathways can be visualized due to their fluorescence nature. Hence, these actively replace conventional fluorescent dyes. Owning to this property, Hailing *et al.* utilized the hydrothermal method for the synthesis of CDs. The glycerol and PEI-25K was used as precursor, while the surface was coated with polyethyleneimine to incorporate the anticancer drug over the outer layer of the CDs through electrostatic interactions. Hep3B and MHCC-97L cells were used to study the drug release, which confirms that the cell death rate increased in the cancer cells compared to the normal cells and showed strong inhibition against tumor growth [134].

Chung *et al.* [135] have synthesized monodispersive CD-hydroxyapatite (HAP) with sugarcane waste and sodium hydroxide via the hydrothermal procedure. The shape of the prepared CD was spherical. The obtained fluorescent CDs were incorporated with the HAP, which is used as a biomaterial to promote tissue regeneration. These CD-HAP enhance the loading efficiency twice as compared to the normal carrier. The release profile was studied by the absorbance of the drug in phosphate buffer saline. The results show that the drug follows the diffusion mechanism, enhancing therapeutic efficacy [135].

Sarkar *et al.* [136] synthesized fluorescence CDs using aloe vera gel and chemically cross-linked them with polymeric hydrogels for controlled drug delivery of vancomycin, a tricyclic glycopeptide antibiotic to the gastrointestinal tract. The synthesized CDs were further coated onto the surface of alginate film through the casting technique to improve thermal stability. The surface modifications on CDs help to enhance more loading capacity of the drug onto the surface. The drug ß-vancomycin coated on CDs was released through the pH-response in pH 1.5, similar to the stomach. The potential uptake of the drug was recorded as 96 %, whereas the release rate was recorded as 56 % in 120 h. This confirms that ß-CDs have great potential as a drug delivery vehicle for oral administration [136].

### Tumor theranostics

Coupling therapeutic agents with ligands complementary to target biomarkers of the diseased site resulted in image-guided therapy to treat the condition better. Adjuvant therapies like PTT, and PDT combined with an imaging system provide information about the size and site of the tumor but also engage in real-time monitoring of the therapy (Table 4). This type of monitoring of the therapeutic effect in the region will enable physicians to plan therapy better with increased therapeutic efficiency. Decorating the surface of CDs with appropriate functional groups will facilitate drug delivery at the targeted site. Due to the significant fluorescence property of CDs, they help to image the same. Further, the CDs can be modified to respond to external and internal stimuli, paving the way for more targeted delivery. This way, CDs eliminate most of the adverse effects and are standalone nanoparticles. Chen *et al.* [137] developed CDs that could respond to dual stimuli and release the drug. CDs were developed via the microwave-mediated method. The prepared particle showed redox and pH sensitivity. Mesoporous silica nanoparticles/CD functionalized with multiple functional groups was developed as the carrier and loaded with DOX as a sample drug. The comparison was made with the carrier with and without cargo.

The CDs produced in the work showed NIR fluorescence that served as the probe for fluorescence imaging, where the surface functional groups provided targeted drug delivery leading to theranostic applications. The silica nanoparticles were used to provide biocompatibility [137]. Luo *et al.* [138] designed a nanohybrid system with CDs doped with iron through a solvothermal approach for tri-therapy with multimodal imaging. The particle was PEGlyated to improve their biocompatibility. The *in vivo* and *in vitro* study against the breast cancer model showed an increased tumor inhibition growth rate. The particle delivered the gene to the site through a photothermal mediated response, and the efficiency of this procedure conducted *in vivo* was around 6-fold higher [138]. Fahmi *et al.* [139] synthesized CDs through a pyrolysis technique coated with magnetite and loaded with naproxen as a model drug. The drug release was found to be pH responsive. The cellular toxicity study done with breast cancer cell lines *in vitro* showed minimal toxic effects, and the delivery of the drug was targeted to cancerous cells alone. This way, the designed hybrid material showed effective theranostic applications [139].

## Conclusion and future perspective

Carbon Dots (CDs) have gained enormous attention in biology and the healthcare industry due to their good aqueous solubility, biocompatibility, inexpensive fabrication procedures, and excellent photoluminescence properties. Due to optical properties and structure-specific properties, CDs possess tunable surface chemistry. Biocompatible CDs were synthesized via various synthetic procedures, such as bottom-up and top-down procedures, and were discussed from small organic molecules and their applications in various fields. Although various methods are proposed for synthesizing CDs, we still lack an understanding of the properties and mechanism to obtain a well-defined structure and uniform size distribution, which plays a key role in deciding the toxicity and fluorescence properties that further affect biological applications. The hydrothermal synthesis approach of synthesizing carbon dots comes out with a well-defined size and structure with functional modification, which helps obtain a high quantum yield and desired photoluminescence properties. However, future research will still be required to gain a thorough understanding of the various fields where CDs can have a significant impact and benefits. As the first step, the fabrication process of CDs needs to be studied since the size of the particles accounts for their fluorescence performance. Control over their size, morphology, and surface properties is crucial to get desired product. The product yield is another concern during the synthesis procedure. Carbon dots prepared especially via the green-mediated strategies exhibit low product yield. Regardless of the issues, CDs still hold a promising position in healthcare, as a little more understanding could resolve most concerns.

## Figures and Tables

**Table 1.** Summary of top-down approaches used for the preparation of CDs for various biomedical applications

Carbon source/ Precursor	Method	Size, nm	Emission / Excitation, nm	Quantum yield, %	Application	Ref.
Histidine hydrochloride	Electrochemical	3-5	505/420	33.8	Selective and sensitive for quantitation of Cu^2+^ ions	[37]
Acetonitrile/BMIMPF_6_*	Electrochemical	~3	422/365	13.3	Imaging of cells and detection of ferric ion	[38]
Sodium citrate and urea	Electrochemical	1-3.5	433/351	11.9	Sensing of Hg^2+^	[39]
Toluene	Laser ablation	4.2	475/330	18	Monitoring fluorescence change to enable real-time analysis	[31]
Graphite and acetone (liquid medium)	Laser ablation	4-20	520/440	-	Biomedical applications, due to its tunable photoluminescence properties	[16]
Graphite flakes	Laser ablation	3-13	430/365	12.2	-	[40]
Polyethylene glycol (PEG)	Sonochemical process- Ultrasonic irradiation	2-7	460/370	~16	Promising in bioimaging and solar cell applications	[33]
Glucose	Ultrasonic treatment	<5	450/350	7	Potential candidate in fluorescence markers, bio-sensors, drug delivery, and biomedical imaging.	[35]
Ascorbic acid and ammonia	Ultrasonic treatment	10	514/385	-	To replace the current self-assembly device and its technology	[41]
Gelatin	Ultrasonic treatment	3.8	450/365	33.8	Cellular imaging and anti-counterfeiting ink	[42]
Graphite	Oxidative cleavage	1.4-4.2	500/350	-	Photodetector device	[43]

*BMIMPF_6_ - 1-butyl-3-methylimidazolium hexafluorophosphate

**Table 2.** Summary of Bottom-up approaches used for the preparation of CDs for various biomedical applications

Carbon source (precursor)	Method	Size, nm	Emission/Excitation, nm	Quantum yield, %	Application	Ref.
Lactose, HCL	Microwave	10	450/350	-	Chemical sensing of heterocyclic aromatic amines	[46]
Glucose, Glutamine and Imidazolium	Microwave	7-15	-	-	Efficient metal-free catalyst in the production of H_2_O_2._	[59]
Citric acid	Microwave	1.5-4.5	530/450	-	Antimicrobial photodynamic therapeutic applications	[60]
Citric acid and diethylenetriamine (DETA)	Thermal decomposition	3-5.5	456/360	88.6	Fluorescent chemosensor- detection of Chromium (VI)	[61]
Dicyanamide (DCD) and citric acid	Thermal decomposition	8-16	528/410	73.2	Detection of iron and fluorine ions	[62]
Citric acid and AEAPMS	Thermal decomposition	1.4	450/360	47	For bioimaging and biosensing applications	[63]
L-DOPA and N, N- dimethylformamide	Carbonization	3.27	400/320	48	Used as a probe for the live cell imaging	[64]
Glucose	Carbonization	1-7	498/400	48	Turn-on biosensor for ovalbumin and *in vivo* imaging applications.	[65]
Sago waste	Pyrolysis	37	390/315	-	Probe for heavy metal detection	[66]
N-hydroxysuccinimide	Pyrolysis	20-30	420/350	14-31	Used in sensors and high-performance optoelectronic devices	[67]
Forestry lignocellulosic waste	Pyrolysis	20-80	438-473/ 350	28	Sensor and contrast agent	[68]

**Table 3.** Summary of the hydrothermal method used for the preparation of CDs for various biomedical applications

Carbon source (Precursor)	Size, nm	Emission / Excitation, nm	Quantum yield, %	Application	Ref.
Citric acid, ethylenediamine, and methyl blue	1.86	440/350	68.0	Fluorescent probes for sensing mercuric ions and chlorine monoxide ions in tap water samples	[93]
Glucose, *m*-phenylenediamine	8	460/320	17.5	Good candidates for fluorescent probes of Fe^3+^, CrO_4-2_ and as cell labeling reagent	[94]
*Thelephora ganbajun Zang*	1.6-7.5	444/356	9.44	Environmental pollutant detection- 2,4-dinitrophenol(2,4-DNP), and 4-nitrophenol (4-NP) in water and soil samples	[95]
Liquid products of biodegradation of coal (LPBC)	3.5	442/328	0.54	pH and temperature-based sensing applications.	[96]
Cysteine, trisodium citrate dehydrate, and ethylenediamine	3.82	442/350	66	Bioimaging of human ovarian cancer cells and fluorescent determination of baicalein	[97]
*Salvia hispanica* L. (Chia)	5.4	415/310	17.8	Targeted treatment of kidney-related diseases	[98]
Persimmon peels	2	350/256	-	-	[99]
Folic acid, glycerol	5.1	442/365	25.3	Detection of Cu^2+^ in water samples	[100]
Citric acid and L-histidine	3.9	414/340	22	Used to analyze chlorogenic acid in coffee and honeysuckle	[101]
Gardenia fruit	2.1	450/360	10.7	Detection of Hg2+ and cysteine	[102]
Sodium citrate, triethylenetetramine, rose Bengal	-	525/440	-	Chemosensors for hypochlorous acid detection	[103]
Citric acid monohydrate, 2-aminopyridine	9	421/310	18	Bioimaging of Candida albicans	[104]
L-Lysine, thiourea	6.86	365/300	53.19	Detection of picric acid in water	[105]
*p*-phenylenediamine	3.2	580/520	-	A strong antibacterial agent inhibits the growth of *S. aureus* and *E. coli*	[106]
Jackfruit peel and tamarind peel	5.3	430/350	13.04	Multifunctional ability, including anticancer activity	[107]
Diethylenetriamine and trans-aconitic acid	2-8	435/365	81	Highly selective and sensitive towards Fe^3+^	[108]
Highland barley	5.8	480/440	14.4	Used for detection of Hg^2+^	[109]
Biomass of *Dunaliella salina*	4.7	415/340	8	Used for intracellular detection of Hg (II) and Cr (VI)	[110]
Palm kernel shells, L-phenylalanine	2	430/360	13.7	-	[111]
L-arginine, phosphoric acid	2.4	444/340	18.38	Efficient fluorescence sensor for detection of vitamin B_12_	[112]
O-phenylenediamine and semicarbazide	4.71	625/540	23	Optoelectronics and forensic science	[113]

**Table 4.** Summary of carbon dots prepared for theranostic tumor applications

No.	Carbon source (Precursor)	Synthesis strategy	Doping	Cargo	Imaging modality	Therapy	Outcome	Ref.
1.	EDTA	Hydrothermal technique	Nitrogen and nickel	-	MRI and Photoacoustic and Photothermal imaging	PTT	The as-prepared CDs served as a probe for multimodal imaging and PTT agent for effective therapy against U14 tumor in a mouse model. The experiments showed that the particle was able to induce potent therapy against the tested cancer model. The particle possessed renal clearance, which could eliminate other long-term toxicity. This way, the particle assured biosafety inside a bodily system.	[140]
2.	*p*-phenylenediamine	Solvothermal method	-	-	Fluorescence and MR imaging	Chemodynamic therapy	The CDs was decorated with gadolinium and ferrous ions to obtain imaging capabilities. The particle was able to emit red fluorescence, which helped in fluorescence imaging. The ferrous ions helped to perform chemodynamic therapy by getting released at the tumor site. This was accomplished through a Fenton reaction. The gadolinium ions were added to perform MR imaging. The study results revealed that the particle featured less toxicity and performed synergistic activity in the management of cancer.	[141]
3.	Citric acid	Hydrothermal approach	-	Photosensitizer- chlorine and copper ions	Fluorescence imaging	PDT, PTT, and Chemodynamic therapy	The copper ions in the formulation helped to perform chemodynamic therapy, while the photosensitizer helped in PDT. The copper ions also achieved GSH depletion resulting in redox-responsive delivery. The *in vivo* and *in vitro* results indicated that the formulation performed FL-mediated tumor therapy.	[142]
4.	Aminosalicylic acid	Hydrothermal method	-	Doxorubicin	Fluorescence imaging	Chemotherapy	A yellow emissive CDs was obtained hydrothermally. The CDs was loaded with anticancer drug and coated with cancer cell membrane. This coating helped to escape immune cells such as macrophages and also to target the cells. The formulation exhibited redox sensitive drug release also. The results from the experiments showed an effective image-guided therapy against cancer.	[143]
5.	Citric acid	Solvothermal method	Gadolinium	AS1411 aptamers	Fluorescence and MR imaging	PTT	The aptamer-conjugated CD nanostructure exhibited the emission of red fluorescence. The doping material made the nanostructure as an imaging probe suitable for MR and fluorescence imaging. The particle also performed PTT due to its photothermal conversion ability.	[144]
6.	Cyanine dye	Solvothermal process	-	-	Fluorescence imaging	PTT	The *in vivo* and *in vitro* study results showed that the particle synthesized possessed high photostability.	[145]
7.	DHCA and EDA	Hydrothermal method	Gadolinium	Doxorubicin and IR825	MR imaging	Chemotherapy and PTT	The as-synthesized CDs emit blue fluorescence. The doping material made the formulation serve as a probe for MR imaging. The *in vivo* experiments conducted on breast cancer-induced mice model showed excellent PTT and chemotherapeutic effect with good tumor inhibition rate.	[146]
8.	*o*-phenylenediamine and lysine	Hydrothermal method	N and S	-	TP fluorescence imaging	PTT and PDT	The study compared the CDs with different doping for the treatment and management of cancer. The results suggest doping of CDs with N and S yields better therapeutic characteristics compared to the CDs doped with N atoms alone. The efficiency of oxygen-producing ability and photothermal conversion of the CDs co-doped with N and S atoms also was comparatively high, providing better theranostic efficiency.	[147]
9.	Polydopamine and folic acid	Hydrothermal method	N doping	-	PSMA-directed fluorescence imaging	PTT	The as-synthesized particle showed good photothermal conversion ability and helped to perform photothermal therapy. The therapeutic approach was mediated via temperature-induced apoptosis. The *in vitro* study with various cell lines indicated that the N-doped CDs functionalized with folic acid ligands induced high cytotoxicity to the prostate cancer cells.	[148]
10.	Watermelon juice	Hydrothermal method	-	-	Optical imaging	PTT	Upon irradiation with NIR laser, the CDs prepared exhibited excellent photothermal conversion efficiency and executed photothermal therapy against cancer *in vivo*. The superior optical properties of the obtained CDs made synergistic optical imaging possible.	[149]
